# Lipid–Protein and Protein–Protein Interactions in the Pulmonary Surfactant System and Their Role in Lung Homeostasis

**DOI:** 10.3390/ijms21103708

**Published:** 2020-05-25

**Authors:** Olga Cañadas, Bárbara Olmeda, Alejandro Alonso, Jesús Pérez-Gil

**Affiliations:** 1 Departament of Biochemistry and Molecular Biology, Faculty of Biology, Complutense University, 28040 Madrid, Spain; ocanadas@quim.ucm.es (O.C.); barbara_olmeda@bio.ucm.es (B.O.); alejaalo@ucm.es (A.A.); 2 Research Institut “Hospital Doce de Octubre (imasdoce)”, 28040 Madrid, Spain

**Keywords:** pulmonary surfactant film, surfactant metabolism, surface tension, respiratory air–liquid interface, inflammation, antimicrobial activity, apoptosis, efferocytosis, tissue repair

## Abstract

Pulmonary surfactant is a lipid/protein complex synthesized by the alveolar epithelium and secreted into the airspaces, where it coats and protects the large respiratory air–liquid interface. Surfactant, assembled as a complex network of membranous structures, integrates elements in charge of reducing surface tension to a minimum along the breathing cycle, thus maintaining a large surface open to gas exchange and also protecting the lung and the body from the entrance of a myriad of potentially pathogenic entities. Different molecules in the surfactant establish a multivalent crosstalk with the epithelium, the immune system and the lung microbiota, constituting a crucial platform to sustain homeostasis, under health and disease. This review summarizes some of the most important molecules and interactions within lung surfactant and how multiple lipid–protein and protein–protein interactions contribute to the proper maintenance of an operative respiratory surface.

## 1. Introduction

In the lung, the essential function of respiration takes place through gas exchange between air and blood. To allow this, a perfect architecture has been developed in which alveoli constitute the functional units of the pulmonary system. There, a thin exchange surface structure is needed to ensure an effective diffusion of oxygen to the capillaries, which is achieved by the existence of flattened alveolar epithelial type I cells (AE1C) that constitute 95% of the total alveolar area [1]. On the other hand, the presence of an air–water interface physically requires a reduction of the surface tension generated by the cohesive forces between liquid molecules that, otherwise, would lead to shrinkage of alveoli [2]. To fulfill this crucial role, alveolar epithelial type II cells (AE2C) secrete pulmonary surfactant, a lipid–protein complex that forms a surface active film at the respiratory interface, allowing its stability and avoiding alveolar collapse during expiration [3,4]. Moreover, beyond this main function, the huge respiratory area exposed to external potentially harmful particles and microorganisms, entails an important role of the lung in protection against pathogens, for which alveolar macrophages (AM) are critical, together with the first line of defense constituted by surfactant [5].

Pulmonary surfactant is mainly composed of lipids (approximately 90% by mass), with phospholipids constituting the principal class, with phosphatidylcholine (PC) as the predominant species (70–80%). Among these, the saturated dipalmitoylphosphatidylcholine (DPPC) accounts for 40% in average (although it can be as much as 50–55% of surfactant mass in humans, pigs, rats or mice), whereas unsaturated PC and anionic phospholipids as phosphatidylglycerol (PG, 8%) and phosphatidylinositol (PI) are also important components. Surfactant also contains neutral lipids as cholesterol, which represents approximately 5–8% in weight. Other less abundant phospholipids include phosphatidylethanolamine, phosphatidylserine and sphyngomyelin, as well as traces of triglycerides and fatty acids [2,6]. Besides lipids, surfactant contains proteins (approximately 10%) from which those surfactant-specific proteins (6–8%) play essential roles to sustain the proper structure and function of the system. SP-B and SP-C are small highly hydrophobic and cationic proteins required to perform optimal surfactant biophysical properties, that is, formation and stabilization of the interfacial film during respiratory dynamics [7,8,9,10]. On the other hand, hydrophilic proteins SP-A and SP-D belong to the collectin (C-type collagen-containing lectins) family, characterized by its ability to recognize and bind to pathogens, providing these proteins with a lung-defense related role [11].

Surfactant lipids and proteins are synthesized in AE2C and assembled into highly packed membranous organelles called lamellar bodies (LB), which are then secreted to the thin fluid phase that lines alveolar spaces [12,13]. Surfactant membranes quickly adsorb to the air–liquid interface to form the functional film that is able to achieve minimal surface tension values upon the reduction of surface area that occurs at expiration, as well as allowing an effective respreading of material during alveolar expansion at inspiration [2]. The finely tuned composition and structure of surfactant complexes are responsible for providing its stabilizing and dynamic properties, so that the presence of certain lipids and proteins (mainly SP-B and SP-C), as well as the formation of a surface-associated multilayer reservoir, are essential for the optimal function of surfactant. Once material is somehow spent and detaches from the interfacial film, recycling (by AE2C) or degradation (by AE2C and AM) take place by regulated routes, ensuring an appropriate amount of operative surfactant at the alveolar spaces [14].

Maintaining lung homeostasis is crucial, including a tight regulation of many different processes occurring at the alveolar and perialveolar environments. Perturbation of any of these regulatory mechanisms entails lung injury and several pulmonary pathologies [13]. First, a balance must be accomplished between inflammatory responses necessary to defend the lung against toxins or pathogens and the need of a basal toleration to avoid exacerbated reactions [15]. Besides this immune homeostasis, ensuring the availability of a proper amount of surfactant with accurate composition and structure, under several physiological or pathological situations, is essential for stabilizing the respiratory surface and supporting alveolar homeostasis [16]. In this review we describe the central role of pulmonary surfactant in different aspects of lung homeostasis, highlighting the importance of lipid–protein and protein–protein interactions in achieving this vital function.

## 2. The Essential Role of Pulmonary Surfactant at the Lung Interface

### 2.1. Formation, Stability and Dynamics of the Surfactant Film: Protein and Lipid Interaction

**Figure 1:**
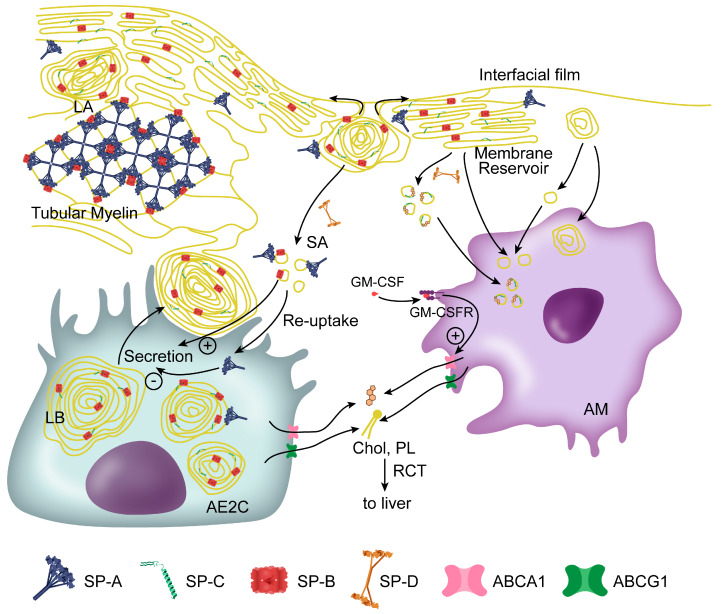
Protein/protein and protein/lipid interactions are essential for maintaining surfactant homeostasis at the alveolar spaces. Surfactant components are synthesized by AE2C and assembled in lamellar bodies (LB). Proper formation of these highly packed lipid organelles requires the presence of SP-B and ABCA3 [20,21]. Upon secretion to the alveolar fluid, surfactant is efficiently adsorbed into the air–liquid interface, in a process highly dependent on the presence of proteins SP-B and SP-C [22,23]. Multilayered membranous surfactant structures associate to the interfacial film forming a reservoir, which provides mechanical stability and a source of material upon respiratory dynamics [19]. Extracellular surfactant structures include a membrane network promoted by SP-A and SP-B (tubular myelin) [24] and membrane arrangements of different complexity [25]. After exposure to air, surfactant “used” membranes are converted into small vesicles [26] in a SP-D-mediated process [27] that can be at least partly re-uptaken by AE2C, a process promoted by SP-A [28]. SP-A also acts as a secretion inhibitory signal [29], in an opposing role to that of SP-B as secretion inducer [30]. Components captured by AE2C are routed to the recycling pathway or degraded [14]. Besides, alveolar macrophages account for a 20% of surfactant clearance [31], for which the presence of granulocyte-macrophage colony stimulating factor (GM-CSF) is required [32]. Alveolar homeostasis highly depends on the effective cross-talk between alveolar macrophages (AM) and AE2C and the maintenance of lipid homeostasis in both cells, involving a proper reverse cholesterol transport (RCT) mediated by ABCA1 and ABCG1 lipid transporters [33,34,35]. SP-A is represented as its most abundant octadecameric form; SP-B as double rings, each formed by six dimers; SP-C as monomers; SP-D as dodecamers.

**Figure 2:**
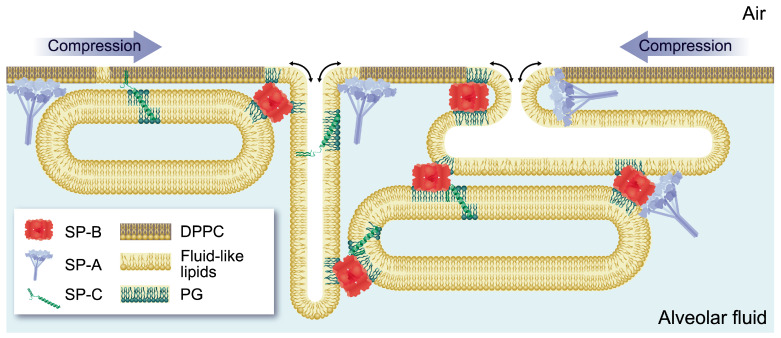
Lipid–protein interactions in the mechanical stabilization of alveoli during breathing cycles. Surfactant proteins SP-B and SP-C facilitate surfactant dynamics by regulating the mechanical properties of surfactant membranes and films through direct interaction with fluid-like lipids [36], including PG. Binding of SP-B and SP-C to unsaturated and anionic lipids would produce protein partitioning into fluid domains that may contribute to highly curved membranes [37], promoting lipid polymorphism [38,39,40,41,42] and favoring a compression-driven enrichment of the interfacial film in the most surface active component of surfactant, DPPC. On the other hand, by binding to DPPC at solid/fluid phase interfaces, SP-A promotes demixing of surfactant lipids, facilitating the segregation of unsaturated phospholipids at the interface [43] and, thus, modeling the mechanical properties of the surfactant film [44]. In addition, surfactant proteins A, B and C stabilize multilayered interfacial structures and preclude the out-of-plane relaxation of the surfactant film at the end of expiration by promoting membrane-membrane contacts: SP-A (oligomer shown, octadecamer) binds simultaneously to different membranes through its carbohydrate recognition domains [10]; SP-B forms rings (shown as hexamers of dimers) and tubes that connect different bilayers [45,46], and SP-C maintains insertion of palmitoylated cysteins at its N-terminal segment into highly packed liquid-ordered regions of the interfacial film [47,48]. Surfactant membranes may be further stabilized during the breathing cycles by SP-A/SP-B and SP-B/SP-C interactions. Notice that surfactant proteins in the cartoon are not represented at equivalent scale.

Upon secretion of lamellar bodies from AE2C into the alveolar liquid subphase, surfactant unpacks and quickly adsorbs to the air–liquid interface forming the surface-active film (Figure 1). This essential ability, together with the reduction of surface tension to very low values during compression of the alveolus and the efficient re-extension of material during inspiration, are strictly required for an effective biophysical function of surfactant, and rely on its particular lipid–protein composition. The saturated acyl chains of the main surfactant phospholipid DPPC are essential to allow for maximal packing of the interfacial film upon reduction of surface area at expiration, thus, reducing surface tension to extremely low values. However, DPPC is not able to efficiently adsorb to the air-water interface by its own, so that anionic and unsaturated phospholipids, together with surfactant proteins, play an essential role in this process [17]. These surfactant components are also required for the generation of a multilayer reservoir of material that is formed during compression of the surface film, once the most fluid regions of the film are forced to fold, leading as a consequence to an enrichment of the air-exposed monolayer in highly packed DPPC-enriched domains (Figure 2). This reservoir of associated membranes, which remains attached to the interfacial film and also incorporates newly arriving surfactant complexes, provides stability and allows fast re-extension of material into the increasing surface area during inspiration [18,19].

Hydrophobic proteins SP-B and SP-C are required for efficient surfactant unpacking upon secretion, and for the formation of the surface film [22]. SP-B in particular plays a vital function in surfactant biophysics. This protein, which belongs to the saposin family, shows lipid membrane fusion and lysis activities that have been ascribed to the different segments of the sequence [49,50,51]. The lipid–protein and protein–protein interactions behind these SP-B properties are responsible for its role in promoting efficient surfactant adsorption and re-extension as well as in maintaining film stability at low surface tensions [17,23]. SP-B is known to be able to generate contacts among membranes [52], and we have shown that SP-B is able to oligomerize into ring structures formed by association of dimers, with a hydrophobic cavity inside [53]. Docking of SP-B oligomers would allow the connection of contiguous surfactant structures and the generation of a hydrophobic tunnel through which lipids could rapidly be transferred across the alveolar fluid phase, adsorbing towards the interfacial film [45,46]. The cohesivity among surfactant membranes provided by SP-B protein–protein interactions would also be important for supporting film stability [54]. Besides offering a hydrophobic pocket, the orientation of the amphipatic α-helices of SP-B at the surface of surfactant layers allows electrostatic interaction of positively-charged residues at the protein with the polar heads of surfactant anionic phospholipids, mainly PG. Within the literature, there is some evidence suggesting a preferential interaction of SP-B and SP-C with anionic phospholipids [45,55,56,57]. This PG/SP-B interaction could be important for the orientation of the protein in the membrane. As a matter of fact, the effect of PG to facilitate surfactant adsorption and in maintaining the stability of the film, was early related to its interaction with cationic hydrophobic proteins, and in particular, with SP-B [58]. The possibility that SP-B oligomer could form a proteolipidic pore implies also the involvement of phospholipids, especially PG, as essential determinants of lipid transfer/adsorption properties of surfactant [45,53,59]. Apart from PG, unsaturated phospholipids play also a role in favoring surfactant adsorption, facilitating a dynamic insertion of the material into the interfacial film [60]. On the other hand, molecular dynamics simulations have very recently proposed potential selective interactions of SP-B with cholesterol, an interesting possibility whose functional implications have still to be clarified [45]. Protein/protein and lipid/protein interactions involved in the formation, stability and dynamics of the surfactant film are summarized in Table 1.

Regarding SP-C, beyond its implication in promoting surfactant adsorption [22,61], it has been ascribed a role in maintaining attachment of the surfactant reservoir to the interfacial monolayer, thus providing stability [62,63,64,65]. A potential cooperation between SP-B and SP-C in biophysical functions in surfactant could be inferred from several studies, including their synergic action in permeability and lipid transfer properties of surfactant [22,23,59,66]. As a matter of fact, a surfactant containing both proteins, SP-B and SP-C, exhibits superior functional properties to facilitate breathing, as tested in vivo, than surfactant preparations lacking one of the hydrophobic proteins [64,67]. Thus, the existence of direct interactions between both proteins has been considered as an interesting possibility, which finally was recently demonstrated by advanced fluorescence spectroscopy techniques [68]. A model has been proposed in which SP-C could also regulate the interaction between SP-B oligomers. The specific and opposing roles of both hydrophobic proteins is revealed by the destabilizing effect of SP-C upon lipid vesicles versus the fusogenic activity of SP-B, and might have functional implications along the surfactant cycle [66,69]. Concerning the preferential interactions of SP-C with lipids, besides PG the protein has been proposed to specifically interact with cholesterol [70]. This neutral lipid is important for maintaining a proper fluidity and viscosity in surfactant, thus facilitating the dynamical properties of the membranes [71,72,73]. However, cholesterol proportion in surfactant must be tightly controlled, so that its increase has been related to functional inhibition [74,75,76]. The presence of SP-C seems to be critical to ensure the proper function of surfactant in the presence of cholesterol [47,77,78], and the combined action of SP-B and SP-C could be important for the removal of cholesterol during film refining at expiration [69].

Despite hydrophobic proteins are the principal players in the biophysical activities of surfactant, the hydrophilic SP-A is also important for an optimal function. This protein, which forms oligomers consisting of six trimers, is able to bind DPPC and cholesterol through its carbohydrate recognition domain (CRD) [79]. SP-A induces calcium-dependent vesicle aggregation and enhanced surface adsorption [80,81], requiring for it a supratrimeric assembly [82]. The interaction of the protein with cholesterol could be involved in the protecting role of SP-A against the surfactant dysfunction produced as a consequence of supraphysiological concentrations of this neutral lipid [83]. Interestingly, slightly different biophysical activity has been found in the two proteins encoded by the two SP-A-related human genes, SP-A1 and SP-A2, which differ in several features of its structure [84,85]. In particular, SP-A1 has been shown to be important for the efficient adsorption of surfactant phospholipids and for the reorganization of the surface film during breathing-like compression-expansion cycles. This role could be related to the high level of oligomerization of SP-A1, which could assist in the formation of the highly cohesive multilayer film, for which SP-B is essential [43]. The existence of recently found protein–protein interactions between SP-A and SP-B [86] would also support the concerted action of both proteins in surfactant processes, and some evidences even suggest the possibility of different modes of interaction of SP-A1 and SP-A2 with SP-B [87], although precise information is still lacking. Thus, protein–protein cooperation could be crucial for developing SP-A physiological roles in surfactant at different levels, including enhancing surfactant adsorption, optimizing film stability and allowing the formation of specific surfactant structures at the alveolar spaces, as tubular myelin [10].

Finally, SP-D, which is not involved in the biophysical activity of surfactant, is not lipid-associated although preferential binding to PI has been proposed, with potential implications for the regulation of surfactant homeostasis, as will be discussed later [88].

### 2.2. Alteration of the Interfacial Surfactant Film is Cause of Lung Injury

As previously described, the surfactant film is essential for reducing surface tension avoiding collapse of alveoli. The presence of surfactant itself is therefore the first indispensable requirement for maintaining lung homeostasis, stabilizing alveoli and preventing edema formation. Besides this, surfactant precludes interfacial stress forces that could deform and cause dysfunction of AE2C as a consequence of their contact with the air–liquid interface [96,97]. This role of surfactant in alveolar micromechanics entails that its deficiency or dysfunction, either by primary (e.g., absence of SP-B, AE2C injury) or secondary (as lung inflammation) causes, triggers lung injury [98,99].

Surfactant deficiency or alteration has been associated with the leading causes of acute respiratory distress syndrome (ARDS), which refers to inflammatory processes frequently associated with lung injury. Inflammation itself mediates AE2C injury, but also liberation of pro-inflammatory mediators and cell and tissue debris, all contributing to an altered composition of surfactant. A complex group of alterations are found in alveolar spaces at ARDS: reduced amount of surfactant lipids and proteins, altered profiles of surfactant protein intermediates and byproducts, increased levels of plasma proteins (because of the increased capillary permeability) that produce surfactant inhibition, increased surfactant hydrolysis of surfactant by secretory phospholipase A2 (PLA2) and release of reactive oxygen species [100,101,102,103,104]. Absence of SP-C and decreased levels of SP-A and SP-B are associated with familial interstitial lung disease [105]. Decreased levels of SP-A and/or SP-D are also observed in neonates with respiratory distress syndrome (RDS) and patients with idiopathic pulmonary fibrosis, bacterial pneumonia, chronic obstructive pulmonary disease, and asthma (reviewed in [106,107,108]). Reduced amounts of lung collectins in the lung increase the susceptibility to respiratory infections, promote inflammation and hamper the elimination of apoptotic cells leading to abnormal lung tissue regeneration as described in the following sections. The finding that elderly people show reduced levels of SP-A in the lungs [109,110] suggests that ageing may aggravate the clinical consequences of surfactant deficiency, particularly in chronic obstructive pulmonary disease and idiopathic pulmonary fibrosis [111]. On the other hand, since lung collectins levels are usually reduced in the lung because of protein leakage towards the vascular compartment, serum levels of these proteins are considered biomarkers of disease severity and predictive factors of unfavorable disease outcome [112,113,114,115].

Genetic deficiency of SP-B is rare, and occurs as a consequence of mutations leading to the absence of SP-B or proSP-B proteins. The disease is characterized by the lack of properly packed lamellar bodies, absence of mature SP-C and appearance of unprocessed proSP-C forms [102,116,117,118]. Unless partial deficiency in SP-B production still allows a certain production of SP-B [119,120], this genetic deficiency gives rise to a lethal RDS upon birth, requiring early lung transplantation to survive [121]. Gene therapy would be thus an essential tool to reverse the usually lethal SP-B deficiency, and promising progress has been recently made in the production of human lung organoids [122]. In the case of adults carrying mutations in heterocygosis, susceptibility to lung diseases in the adulthood has been described, particularly among smokers [123]. Unlike SP-B, genetic SP-C deficiency is not usually a cause of neonatal RDS, but interstitial lung disease, including pulmonary fibrosis, can appear at long-term [124,125,126]. Pathophysiology of the disease caused by SP-C mutations is mainly related to AE2C cell damage, produced by endoplasmic reticulum (ER) stress due to proSP-C misfolding, or to its altered traffic to lamellar bodies [102]. On the other hand, different studies have shown that mutations in the genes encoding SP-A1 and SP-A2 are also involved in lung diseases. For example, mutations that affect the CRD domain of SP-A1 (W211R) or SP-A2 (G231V and F198S) are associated with idiopathic interstitial pneumonia [127] and pulmonary fibrosis [128,129], respectively. These mutations impair protein secretion and promote protein aggregation within the ER, increasing ER stress at resident AE2C [129]. In addition, genetic polymorphisms of lung collectins are known to be associated with susceptibility to acute lung injury [130], RDS [131] and infections [132,133]. In the case of acute lung injury, a polymorphism that causes the insertion or deletion of three amino acids within the collagen-like domain of SP-A1 has been related to the reduced water levels in the lung characteristic of this disease, probably due to alterations in the structure and binding properties of the protein [130]. Complex interactions of single nucleotide polymorphisms of the genes that encode all surfactant proteins seem to contribute to the pulmonary disease in cystic fibrosis patients [134]. Similarly, interactions between SP-A1 and SP-B polymorphisms are involved in genetic susceptibility to RDS [135].

## 3. Surfactant Homeostasis in the Alveolar Spaces

To ensure the presence of a functional surfactant film at the respiratory interface, synthesis, secretion, reuptake and degradation of surfactant components must be perfectly regulated and balanced. This includes achieving both a precise composition of lipids and proteins and their proper organization in defined surfactant structures at the alveolar spaces, together with an efficient cross-talk established between epithelial cells and alveolar macrophages. Protein and lipid interactions involved in surfactant homeostasis are also summarized in Table 1.

### 3.1. Synthesis, Storage and Secretion of Pulmonary Surfactant

De novo synthesis of surfactant phospholipids takes place at the endoplasmic reticulum in AE2C. PC is synthesized from choline by the Kennedy pathway, which includes several enzymatic steps, with the cytidine triphosphate-phosphocoline cytidyltransferase (CCT) controlling the synthesis rate. A parallel formation of diacylglycerols occurs coupled to this pathway (for a recent review on surfactant metabolism see [13]). Direct interaction of acyl-CoA:lysophosphatidylcholine acyltransferase (LPCAT1) with the STAR10 (steroidogenic acute regulatory-related lipid transfer protein 10) protein has been found to be important for trafficking of newly synthesized DPPC from endoplasmic reticulum to LB [136], which seems to occur by direct non vesicular transport between both organelles, apparently skipping Golgi and multivesicular bodies [137]. In fetal mouse lung, glycogen storages present in AE2C, in which CCT is also found, seem to provide substrate for PC biosynthesis [138,139]. Besides this, triglycerides have been also shown to be directed from alveolar lipofibroblasts to AE2C and contribute to the fetal surfactant phospholipid pool, pointing to a cross-talk between these two cell types to ensure surfactant homeostasis [140,141]. Apart from this de novo synthesis, in the Lands cycle DPPC is also obtained by remodeling from unsaturated PC by PLA2 and LPCAT1, being the latter also involved in the synthesis of PG. These two enzymes, together with CCT, have been shown to be key players for the proper regulation of surfactant synthesis, so that their alteration is cause of respiratory distress [142,143,144]. In the case of LPCAT1, it has been ascribed a role in coordinating the two pathways of DPPC synthesis according to the physiological demands of surfactant at the alveolar spaces [145]. Besides this, the finding that peroxiredoxin 6, a multifunctional enzyme with PLA2 and LPCAT activities, is located in lamellar bodies, pointed to these organelles as a sufficient location for DPPC remodeling [146]. Regarding to PLA2, not only this enzyme has a key role in regulating the turnover of surfactant phospholipids because of its implication in their synthesis and degradation, but it is also involved in antioxidant and cell signaling roles [147]. Finally, the interaction of SP-A with peroxiredoxin leads to inhibition of its PLA2 activity, pointing to a potential role of SP-A in regulating surfactant phospholipids turnover [91,148]. With regard to cholesterol, apart from the principal plasmatic source, it can be also synthesized in peroxisomes in AE2C, whereas cholesterol-binding proteins have been found in lamellar bodies, suggesting a potential implication of the Niemann–Pick C pathway in the regulation of surfactant cholesterol and homeostasis [149,150]. Finally, alveolar lipofibroblasts could also participate in cholesterol supply to surfactant under certain conditions [141].

The synthesis and complex processing of proteins SP-B and SP-C initiate in the endoplasmic reticulum of AE2C, and occur coupled to surfactant lipid synthesis and intracellular trafficking. Both proteins are produced as large soluble precursors where the mature hydrophobic modules are protected by flanking N- and C-terminal domains [13]. Processing of precursors takes place in transit to LBs via multivesicular bodies, and consists of several proteolytic cleavages and post-translational modifications, in a route coupled to sequential acidification. Thus, maturation of both proteins is conducted in a concerted way, being SP-B required for proper SP-C processing [117] by mechanisms that are still unknown, although they could well involve the previously described SP-B/SP-C interactions [68], or even potential interactions between its precursors. Furthermore, the fusogenic and lytic properties behind membrane remodeling abilities of SP-B, together with its role in generating membrane contacts, turn SP-B into an essential requirement for the formation of correctly assembled LBs [20,151]. These organelles are formed by packed lipid membranes in a well-defined structure that, in the case of humans, is arranged as concentric lamellae [152]. Surfactant lipids, at least PC, PG and cholesterol, are imported and accumulated into LBs by their limiting membrane transporter ATP binding cassette subfamily A member 3 (ABCA3), in an ATP-depending process [21,153,154]. The coupling between lipid and protein synthesis, processing and assembly in LBs places all these actors as essential providers of surfactant homeostasis, so that their disruption leads to alteration affecting the rest of components of the system and inadequate surfactant production. Thus, absence of CCT is cause of changes in surfactant protein levels and aberrant LBs [142], mutations in ABCA3, or in its interacting protein EMC3 (endoplasmic reticulum membrane protein complex subunit 3), which is required for ABCA3 stabilization, produce absence of lamellar bodies and misprocessed surfactant proteins [155,156], and decrease of SP-B levels affects SP-C processing and LB formation, as previously stated. On the other hand, mutations in the SP-C gene, as previously described, are related to long-term lung diseases, including fibrosis, and injury to AE2C by endoplasmic reticulum stress due to protein misfolding (mutations affecting SP-C precursor) [102]. Finally, absence of ABCA3 has been also related to AE2C apoptosis by endoplasmic reticulum stress due to the accumulation of phospholipids and cholesterol, which are then rerouted to the endoplasmic reticulum from LB [154].

Hydrophilic proteins SP-A and SP-D are synthesized in the endoplasmic reticulum, where post-translational modifications and oligomerization also occurs. The main route of secretion of these proteins is via constitutive non-regulated vesicular transport, bypassing LBs, although a certain proportion of synthesized SP-A seems to be directed to these organelles, together with the recycled protein recovered from alveolar spaces [13]. Due to the role of hydrophilic proteins in lung immunomodulation, their absence produces increased inflammation [157], although alterations in surfactant homeostasis are also found, related to their involvement in surfactant catabolism [90,158], as it will be discussed later.

LBs are secreted to the alveolar fluid in response mainly to alveolar stretching produced during inspiration, through stimulation by purinergic agonists such as ATP. The stimulation of AE2C by ATP leads to an increase in cytoplasmic calcium, both by entry from the alveolar medium and by release from intracellular stores, mainly endoplasmic reticulum. Calcium triggers LB secretion by regulating the direct fusion of LBs with the plasma membrane and by cytoskeleton activation. Generation of the fusion pore, involving soluble SNARE (*N*-ethylmaleimide-sensitive factor attachment receptor) proteins at both the LB and the cell membrane, triggers a local entry of extracellular calcium (fusion-activated calcium entry) that expands the pore and allows surfactant release, which requires compression of the vesicle by an actin coat [13]. Regulation of surfactant secretion is essential to maintain alveolar homeostasis. Extracellular SP-A was described as an inhibitor of secretion by interaction with its receptor tumor protein 63 (P63) at the AE2C membrane [28,29], although little is known about the mechanisms involved in sensing the availability of surfactant at the alveolar spaces and the possible need for secretion of material. Stretching sensing has been traditionally ascribed to AE1C, which would as a result induce LB secretion through the increase of extracellular ATP [159]. However, an implication of the air–liquid interface itself has been reported, so that AE2C could be able to sense the mechanical forces produced by the thinning of the aqueous phase and the increase in surface tension, responding with intracellular calcium increase, thus promoting the secretion of a surplus of new surfactant material. Besides this, AE2C could be also capable of activating cellular responses involved in cell repair, to prevent lung injury [96,97]. On the other hand, extracellular SP-B has been recently found to stimulate surfactant secretion by the purinergic pathway, so that the protein could ensure the availability of enough density of surfactant and a proper membrane network at the alveolar spaces, and thus, surfactant homeostasis [30]. The molecular mechanism of this regulation needs still to be investigated, as well as the potential existence of a “SP-B receptor” at the AE2C plasma membrane. The possibility that SP-B/SP-A interactions [86], as a secretion activator/inhibitor couple, might be involved in a concerted regulation of surfactant secretion will require further investigation. Finally, a potential role as sensor of the surfactant pool size or its composition has been postulated for G-protein coupled receptor 116 (GPR116), a transmembrane receptor of AE2C that has been identified as a negative regulator of surfactant secretion. The sensing mechanism of this protein could rely on its interaction with some surfactant component(s) which, up to now, has not been yet identified [160,161]. However, suggested possibilities for GPR116 sensing include interaction with SP-D or its direct modulation by mechanical stretching upon inspiration [162].

### 3.2. Surfactant Structures in the Alveolar Spaces

Once surfactant is secreted into the alveolar fluid, fast adsorption of material takes place and new components are transferred into the air–liquid film. The specific mechanisms converting the secreted highly packed membrane structures into part of the interfacial film are not still completely understood. SP-B/SP-C common machineries have been shown to be essential for unpacking of secreted surfactant LB-like particles and formation of the surface film, pointing to the possibility that, upon contact with the air-water interface, SP-B nanorings, regulated by SP-C, would trigger the fast transfer of lipids into the film [22]. Apart from these freshly secreted structures, another surfactant assembly that could act as an intermediate between LB and the interfacial film, is tubular myelin (Figure 1). Tubular myelin is a complex three-dimensional membranous tubular structure, whose formation requires the presence of SP-A (in humans, both SP-A1 and SP-A2), SP-B, and anionic lipids, highlighting once again the essential role of the interactions among these surfactant components [24,163,164]. Although the particular function played by tubular myelin is still unknown, as its presence is not essential for respiration [165], potential roles could be related with the involvement of SP-A in antimicrobial defense.

The study of the extracellular surfactant recovered from alveolar spaces led to the finding that two different pools of surfactant are present, in terms of size, composition and structural complexity. The more dense surfactant material known as large aggregates (LA), which contains secreted LBs, tubular myelin and SP-A, SP-B and SP-C, is thought to be somehow metabolically converted into another form rich in small vesicles (small aggregates or SA) with decreased content in proteins and a lower adsorption activity [25,26]. Of note, an increased SA/LA ratio is found in patients suffering ARDS [103]. The conversion of LA to SA can be reproduced in vitro by compression-expansion cycling of the interfacial film, so that it is generally assumed that the smaller structures are generated by the “used” surfactant components once surfactant has been exposed to a dynamic air-water interface (Figure 1). The physiological role of these structural changes of surfactant seems to be encompassed in an important mechanism of surfactant homeostasis regulation, as it will be discussed below.

### 3.3. Surfactant Recycling and Catabolism

The proper regulation of surfactant pool size includes the clearance of the used surfactant from the alveolar spaces, thus avoiding the excessive accumulation of a spent, substantially altered (including extensive oxidation) material that can lead to lung injury and inflammation. Surfactant is removed from the alveolar fluid by re-uptake to AE2C for recycling or degradation, and to alveolar macrophages for its catabolism. Surfactant pool size itself has been shown to induce the increase of catabolic rate, thus maintaining alveolar homeostasis, although the molecular pathways involved in such response are not clear [166].

Apart from inhibiting surfactant secretion, SP-A contributes to alveolar homeostasis by stimulating the phospholipid uptake by AE2C via clathrin-mediated endocytosis upon interaction with its receptor P63 [28]. A similar case is GPR116, which acts both as secretion inhibitor and as stimulator of pneumocyte re-uptake, for which potential interactions with SP-D may be involved [95,162]. As a matter of fact, SP-D seems to play an essential role in the regulation of surfactant pool size. This protein is involved in the structural remodeling of surfactant structure into SA, which is the form taken up by AE2C to proceed with surfactant recycling or degradation (Figure 1). To achieve this role, for which SP-D oligomeric structure is required [167], the protein seems to induce fragmentation of surfactant membranes, for which its interaction with PI has been suggested to be a determinant factor [27,94,168]. On the other hand, the structure of surfactant aggregates does not influence its uptake by AMs [169], and although SP-D is able to bind to the surface of these cells to modulate inflammation, the homeostasis/immune roles of the protein seem to be independently regulated [94]. The regulation of surfactant homeostasis carried out by SP-D is particularly important upon birth. Newborn mice show a much larger pool size and an ultrastructure of SA and LA pools different to that observed in adults. The postnatal change leading to a normalization of the pools has been suggested to be initiated by the increased amount of PI of newly secreted surfactant (both in newborns and adults) that promotes the association of SP-D to surfactant lipids, triggering the structural conversion to small vesicles [93,94]. All these findings highlight how the particular composition and structure of surfactant at the different levels of its functional cycle regulate alveolar homeostasis in detail, and how the interactions between surfactant proteins and lipids are particularly essential for this task.

Maintaining lipid homeostasis inside the cell is crucial both for AE2C and AM, mainly through the ATP-binding cassette transporters ABCA1 and ABCG1. These proteins are involved in the reverse cholesterol transport by mediating efflux of excess cholesterol and phospholipids towards the plasma (Figure 1). In AE2C, alteration of these proteins produces lipid accumulation and affects LB morphology, secretion and recycling, suggesting an important role in surfactant homeostasis [35,170].

Alveolar macrophages are essential for surfactant lipids catabolism, accounting for around 20% of its clearance [31]. Alterations affecting lipid degradation by AM entails accumulation of lipids in these cells (the so-called foamy macrophages) that causes inflammation, and reduced clearance of surfactant, leading to accumulation of impaired surfactant at the alveolar spaces. Factors involved in these pathways, such as cluster of differentiation CD44, are thus regulators of lung homeostasis and inflammation [171]. The main regulator of lipid catabolism in AM has been shown to be granulocyte-macrophage colony stimulating factor (GM-CSF) (Figure 1). This factor does not affect surfactant uptake by AM directly but promotes the maturation of these cells and activates proteins involved in phospholipid and cholesterol clearance, as ABCA1 and ABCG1 [32,34,172]. On the other hand, the administration of GM-CSF to mice with impaired AM development has been recently shown to produce a decrease in the amount of SP-D, pointing to a cross-regulation of AE2C re-uptake and AM catabolic pathways [33]. Surfactant would then mediate somehow the communication between both cells, as it seems to occur also in relation to cholesterol metabolism, so that the increase of cholesterol in surfactant has been described to trigger alteration of cholesterol metabolism in AM [34]. On the other hand, an involvement of SP-B has been suggested in facilitating cholesterol efflux from AM to surfactant, which would act as acceptor with the aim of reducing inflammation-induced cholesterol accumulation in these cells [173]. A potential role of SP-B/cholesterol interactions in this activity has still to be demonstrated. Regarding SP-C, its reported membrane fragmentation ability, together with its tendency to partition into cholesterol-rich environments have been also suggested to be potentially involved in regulatory mechanisms of surfactant homeostasis [69]. Intrinsic absence of SP-C in AM with cholesterol accumulation in SP-C-KO mice leads to an impaired metabolism and the generation of cholesterol crystals in AM cytoplasm, a phenotype that has been suggested to be the consequence of the impairment of AE2C/AM cross-talk, and that could be mediated by the alveolar instability generated by the absence of SP-C [78].

### 3.4. The Importance of Surfactant Anabolism/Catabolism Balance and Its Alteration

An altered lung surfactant homeostasis as a consequence of unbalanced anabolism/catabolism leads to the generation of pulmonary alveolar proteinosis (PAP). PAP is a syndrome characterized by the accumulation of alveolar surfactant and dysfunction of AM, thus producing respiratory deficiency and alteration of the lung immune defense. The existence of such numerous proteins involved in the regulation of lung lipid homeostasis entails a broad etiology for PAP, including alterations of re-uptake/catabolism by AE2C or AM (as autoantibodies to GM-CSF, absence or alteration in GM-CSF receptors colony stimulating factor 2 receptors alpha and beta, ABCA1, ABCG1, SP-D and GPR116) or of surfactant secretion (GPR116). Besides this, mutations in genes required for the proper production of surfactant (ABCA3, SP-B or SP-C) also leads to congenital PAP [16]. Regarding to the nature of the accumulated lipids, the analysis of the lipidome of PAP samples from different etiologies revealed similar results, including drastic increase of sphingolipids (mainly ceramide), free and sterified cholesterol, PC and lysophosphatidylcholine [174].

Alteration of surfactant homeostasis can also occur upon exposure to external toxicity. Electronic cigarettes have been found to alter lipid homeostasis in AE2C and AM, with effects in ABCA1, ABCA3, LB structure and immune functions [175]. Finally, chronic alcohol consumption also disrupts lipid homeostasis and immune responses in AM, increasing susceptibility to pulmonary infections [176].

In conclusion, the importance of a proper lipid homeostasis in the lung goes beyond the necessity of maintaining sufficient but proportionate lipid levels to ensure surfactant function and alveolar stability. Lipids play essential roles in lung physiology, and constitute a link between metabolism and host defense, sensing and translating extracellular information to immune cells. As will be detailed later, anionic phospholipids influence AM response to inflammatory stimuli and oxidized lipids and cholesterol mediate and expand inflammatory responses [177]. Of note is the role of PLA2, involved in surfactant lipid remodeling and degradation, in connecting surfactant turnover and oxidative stress responses in AM [147].

## 4. Anti-/Pro-Inflammatory Balance

The constant exposure of the lung to microorganisms from inhaled air at the upper respiratory tract activates the immune response, which leads to transcriptional expression of inflammatory mediators that coordinate the elimination of infected cells and pathogens. Resolution of inflammation and return to homeostasis is induced by activation and secretion of endogenous anti-inflammatory factors [178]. However, aberrant activation of the inflammatory response leads to immunodeficiency, septic shock, or induction of autoimmunity [179]. Therefore, the activation and resolution of inflammation must be tightly regulated by immune mechanisms that decrease microbial burden by facilitating clearance of inhaled pathogens, and control tissue damage caused by pathogens or by the host immune response by modulating inflammatory responses [180]. Surfactant proteins and lipids have been shown to provide immune protection against respiratory pathogens, both directly by limiting inflammation and promoting pathogen clearance, and indirectly by activating molecular and cellular mechanisms that contribute to restore lung homeostasis [15,181,182,183]. This part of the review summarizes the role of surfactant proteins and lipids on the immune response against bacterial lipopolysaccharide (LPS), the most potent pathogen-associated molecular pattern among bacterial cell wall components. 

### 4.1. Interaction with Bacterial Lipopolysaccharide

**Figure 3:**
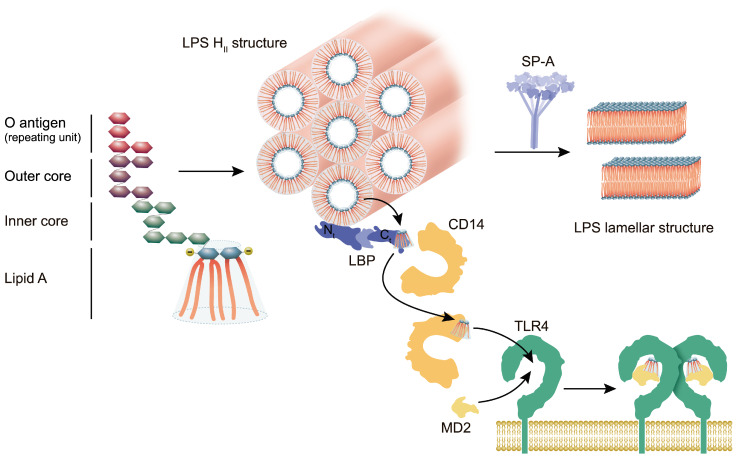
Host response to lipopolysaccharide (LPS). LPS is composed of lipid A, the hydrophobic membrane-anchoring region, and a hydrophilic part formed by the core oligosaccharide region and the O-antigen. The core oligosaccharide region includes an outer core region, enriched in hexoses, and an inner core region proximal to lipid A that contains 3-deoxy-D-*manno*-octulosonic acid [184]. The O-antigen, also termed O-specific chain, contains up to 50 highly variable oligosaccharide units. Both the acyl chains and the negative charges of the phosphate groups bound to the diglucosamine backbone of lipid A are required for the recognition of LPS by cell receptors and the subsequent activation of the host immune system. LPS molecules associate forming non-lamellar supramolecular structures that are recognized by LBP, which transfers LPS monomers to CD14. LPS is subsequently transferred to the TLR4/MD2 complex, triggering complex dimerization and initiating the signaling cascade. Interaction of surfactant protein SP-A (functional form, octadecamer) with LPS modifies the aggregated LPS structure, inducing formation of lamellar phases, and therefore preventing the recognition of LPS by LBP.

Bacterial lipopolysaccharide, the major component of the outer membrane of Gram-negative bacteria, strongly stimulates the innate or natural immunity in eukaryotic species [184]. LPS is composed of a poly- or oligo-saccharide part linked to a membrane-anchoring lipophilic domain known as lipid A or endotoxin (Figure 3). The polysaccharide part is commonly subdivided into the terminal O-specific chain, whose composition varies within bacterial species, and the rather invariable core region, most proximal to lipid A. Bacteria express either smooth LPS, which contains the O-antigen and complete core oligosaccharides, or rough LPS, which lack O-antigen and possess progressively shorter core oligosaccharides. Rough LPS is designated as Ra, Rb, Rc, Rd, and Re in order of decreasing core length [185]. Strains with rough type LPS are more common among pathogens colonizing pulmonary compartments [184,186,187].

Lipid A, which represents the conserved molecular pattern of LPS, consists of a β(1→6)-linked diglucosamine backbone carrying two phosphate groups at positions 1 and 4’ of the glucosamine residues and six or seven ester- and amide-linked saturated acyl chains of 12 and mostly 14 carbon atoms in length [184] (Figure 3). Both phosphate groups and the myristoyl and lauroyl acyl chains have been shown to be major determinants for specific recognition and activation of innate immunity. LPS recognition by cell receptors requires a specific three-dimensional non-lamellar supramolecular structure of LPS aggregates [185,188,189,190] (Figure 3).

Host responses to LPS are mainly mediated by the pattern recognition receptor Toll-like receptor 4 (TLR4) [191]. The activation of TLR4 by LPS requires the interaction of LPS with several receptor-proximal proteins (Figure 3). The lipopolysaccharide binding protein (LBP) binds to the aggregated structures of LPS and transfers LPS monomers to the soluble or membrane-bound forms of cluster of differentiation 14 (CD14) via specific recognition of the lipid A domain. CD14 subsequently transfers LPS to the myeloid differentiation factor 2 (MD2), which forms a complex with TLR4. LPS binding to MD2 induces a conformational change in TLR4 that promotes TLR4/MD2 dimerization and the subsequent CD14-controlled internalization of TLR4 into endosomes [192], initiating the signalling cascade [193]. Using different LPS analogs, Triantafilou and co-workers [194] showed that the recruitment of these receptors is triggered by the 1-phosphoryl lipid A moiety of LPS.

Surfactant proteins -A, -C and -D have been shown to act as LPS scavengers both in vitro and in vivo [195,196,197], contributing to the clearance and inactivation of LPS released by Gram-negative bacteria. These proteins interact with different LPSs [198,199,200,201,202,203]. While SP-D binds to the acyl chains of lipid A [204] as well as to the heptoses and mannoses of the core [205,206] and O-antigen [207], respectively, SP-A only binds to lipid A [208,209]. As a result, SP-A only binds to rough LPS since the bulky headgroup of smooth LPS hinders the access of SP-A to the lipid A moiety. Although the interaction of SP-A and SP-D with LPS was firstly described as being mediated by calcium [208,209,210], it has been shown that both collectins bind to Re-LPS in the absence of this cation [202,203,211]. On the other hand, SP-C binds to the lipid A moiety of LPS by its N-terminal segment, probably through electrostatic interactions between the polar and basic residues of the protein and the negatively charged terminal 1-phosphate group at the reducing end of the lipid A disaccharide [199]. Similarly, SP-A binding to the 1-phosphate of lipid A [212] may involve electrostatic interactions with a cluster of basic residues located in the CRD of the protein [203,213]. Since the negative charges of the phosphate groups are required for LPS binding to LBP [214] and the activation of the TLR4/MD2 complex [215], the interaction of SP-C and SP-A with 1-phosphate may shield this functional group, blocking some of the physiological responses to LPS in the lungs. This hypothesis is supported by the finding that SP-A prevents the interaction of Re-LPS with LBP [216]. SP-A also agglomerates rough LPS aggregates in the presence of calcium [203] and rearranges the aggregated structure of LPS, which changes from inverted to lamellar phases [212] (Figure 3). Both the clustering of LPS aggregates and their lamellar structure would reduce LPS toxicity by preventing the recognition of LPS by LBP, and the subsequent initiation of the LBP/CD14 pathway towards inflammatory reactions.

Surfactant lipids can facilitate the scavenger action of surfactant proteins. In this regard, it has been shown that LPS incorporates in vesicles of a modified porcine pulmonary surfactant [217] and films of surfactant lipids [196,211]. Repulsive interactions between smooth LPS and surfactant lipids facilitate the interaction of SP-A with the lipid A moiety of smooth LPS, which results in the extraction of LPS molecules [196]. Additionally, incorporation of LPS into surfactant membranes would decrease the endotoxicity of LPS by abrogating the interaction of lipid A with its cellular receptors. This hypothesis is supported by earlier studies reporting that the biological activity of LPS is reduced when LPS is incorporated into liposomes [218]. Alternatively, surfactant lipids may incorporate into LPS aggregates, changing the aggregated structure of LPS, and hence, reducing its ability to stimulate immune cells [190].

### 4.2. Interaction with Pattern-Recognition Receptors

In addition to the direct interaction with LPS, surfactant proteins also modulate the cellular inflammatory response via interaction with pattern-recognition receptors and associated molecules. For instance, SP-A modulates the cellular response to smooth LPS, although this LPS is not a ligand for SP-A, by binding to CD14 [210] and the TLR4/MD2 complex [219]. Thus, the interaction between SP-A and LPS receptors significantly decreases the binding of smooth LPS and consequently prevents the smooth LPS-elicited cellular response [210,219]. In contrast, SP-A significantly enhances the binding of rough LPS to CD14 [210] and does not attenuate rough LPS binding to TLR4/MD2 [219]. This effect has been related to the formation of a SP-A/CD14/rough LPS complex in which the amphipathic neck domain of SP-A would bind to the leucine-rich region of CD14, whereas the CRD would bind to the lipid A moiety of rough LPS, allowing the transfer of LPS molecules to the TLR4/MD2 complex and initiating physiological responses against pathogens [208]. Formation of a ternary complex with CD14 and LPS has also been proposed for SP-C, which, like SP-A, binds to CD14 increasing the binding of rough LPS [220]. SP-C-containing phospholipid vesicles, but not lipid vesicles alone, block LPS-induced cytokine production by a TLR4-dependent mechanism in human embryonic kidney HEK293T cells [197]. Whether this effect is due to the direct interaction of SP-C with TLR4 and/or MD2 needs to be clarified. On the other hand, SP-D binds to CD14 and TLR4/MD2 through its CRD [208,221,222], decreasing the binding of both smooth and rough LPS to CD14 [208] and TLR4/MD2 [223]. This suggests that SP-D may act as a negative regulator to protect against a chronic inflammatory state induced by the release of numerous inflammatory mediators as a consequence of the interaction of SP-A and SP-C with rough LPS and its receptors.

On the other hand, anionic surfactant lipids PG and PI inhibit LPS-induced cell activation by competitive binding to LPS receptors [224,225]. While PI interferes with the interactions between LPS and CD14 [225,226,227], PG inhibits LPS binding to LBP, CD14 and MD2 [190,224,225,228]. Alternatively, surfactant lipids can prevent activation of TLR4 by LPS by incorporating it into the plasma membrane. Thus, DPPC has been shown to inhibit the translocation of TLR4 to raft domains [229], precluding the co-localization of CD14 and TLR4 in lipid rafts that is required for LPS-induced activation of TLR4 [230]. Voelker and co-workers [231] have suggested that inhibition of Toll-like receptor function by surfactant lipids would set a threshold for the engagement of inflammatory cascades in the lung, preventing inflammation by casual environmental stimuli like environmental levels of particulated LPS while allowing a robust inflammatory response during established bacterial infections.

## 5. Antimicrobial Effect

**Figure 4:**
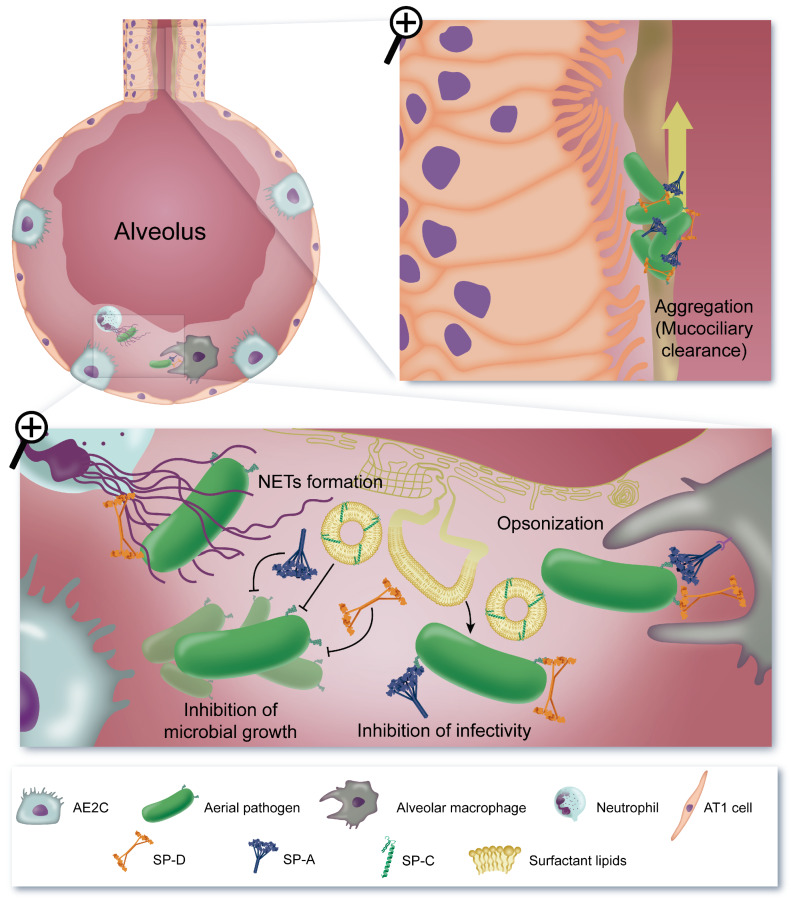
Antimicrobial activity of surfactant lipids and proteins. Lung collectins contribute to microbial clearance by: (i) facilitating mucociliary clearance through microbial aggregation/agglutination; (ii) enhancing microbial phagocytosis via opsonization, upregulation of the expression of cell-surface receptors involved in microbial recognition by phagocytic cells, and regulation of the complement system; and (iii) favoring bacterial trapping by NETs through simultaneous binding to bacteria and NETs. In addition, SP-A (18-mer), SP-C and SP-D (here only 12-mers are represented) directly affect growth and viability of microorganisms by increasing the permeability of the microbial cell membrane. Surfactant lipids and proteins also inhibit microbial infectivity, preventing the adhesion and internalization of bacteria and viruses by alveolar epithelial cells.

Surfactant proteins and lipids also contribute to lung homeostasis by exerting antimicrobial effects (Figure 4). Animal models of SP-C and collectin deficiencies show a significant defect in host defence since: (i) SP-C, SP-A, and SP-D knock-out mice are more susceptible to bacterial, fungal, and viral infections [157,232,233,234,235], (ii) SP-A^−/−^ and SP-D^−/−^ mice show reduced uptake of these microbes by alveolar macrophages (reviewed in [236]), and (iii) bacterial clearance is restored by intratracheal administration of SP-A [237].

Lung collectins SP-A and SP-D prevent microbial dissemination by direct binding to a broad range of microorganisms, including viruses, fungi and Gram-positive and Gram-negative bacteria (reviewed in [236,238,239]). These proteins recognize different pathogen-associated molecular patterns on the surface of microorganisms: LPS in Gram-negative bacteria, lipoteichoic acid and peptidoglycan in Gram-positive bacteria, lipoarabinomanan in mycobacteria, phospholipids in mycoplasma, glucan and mannose in fungi, and glycoproteins in fungi and viruses [236,240,241,242,243,244]. The association of SP-A and -D with pathogens may result in microbial aggregation, which facilitates mucociliary clearance by the respiratory tract, prevents attachment of pathogens to cell surfaces, inhibits microbial colonization and invasion, and may favor microbial phagocytosis [245]. SP-A and SP-D also act as opsonins, increasing the association, uptake and killing of the microorganisms [246]. This requires specific interactions with different receptors on phagocytic cells, such as surfactant protein receptor 210 (SP-R210) [247], a specific receptor for SP-A [248], the putative opsonin receptor glycoprotein 340 (gp340), a macrophage scavenger receptor family member, to which both SP-A and SP-D bind [249,250], or the immunoglobulin G [251]. Interaction of the globular heads of lung collectins with pathogen-associated molecular patterns promotes the presentation of the collagenous tails to calreticulin/cluster of differentiation 91 (CD91) on the surface of phagocytic cells, stimulating phagocytosis and pro-inflammatory responses [252]. Lung collectins can also stimulate phagocytosis through upregulation of the expression of cell-surface receptors involved in microbial recognition in phagocytic cells [253], such as the mannose receptor [254,255] or the class A scavenger receptor [256]. The precise receptor(s) involved in the activation of the signal transduction pathways that lead to an increased trafficking of pathogen recognition receptors to the plasma membrane remains elusive. Alternatively, lung collectins may promote microbial clearance by regulating the complement system. Thus, SP-D enhances the activity of complement component 1q (C1q) by forming additional binding sites for C4b and C3b [257], two complement binding protein complexes involved in opsonization [258]. On the contrary, association of SP-A with C1q may down-regulate complement activity, preventing C1q-mediated complement activation and inflammation [259]. Lung collectins are also involved in pathogen clearance by neutrophil extracellular traps (NETs), DNA-based extracellular traps that capture and kill microbes. Simultaneous binding of SP-D to bacteria and NETs promotes bacterial trapping by the NETs [260]. On the other hand, since LPS efficiently induces the formation of NETs [261], binding of SP-D to LPS has been recently shown to attenuate NETs formation [262]. This suggests that SP-D may regulate the balance between the antimicrobial activity of NETs and the exacerbated immune response and tissue injury as well as surfactant inhibition induced by an excessive presence of NETs in the lung.

SP-A and SP-D directly affect the growth and viability of microorganisms [263]. Lung collectins have been found to inhibit the growth of different bacteria like *Escherichia coli*, *Klebsiella pneumoniae*, *Enterobacter aerogenes*, *Legionella pneumophila*, *Mycobacterium avium*, and *Mycoplasma pneumoniae* [236,240]. This bacteriostatic effect has been related to the ability of SP-A and SP-D to increase the permeability of the microbial cell membrane [264]. Gram-negative bacterial strains decorated with rough LPS are permeabilized more effectively by SP-A and SP-D than strains containing smooth LPS [264]. Although the mechanism of bacterial permeabilization by lung collectins is unclear, it seems to require a direct interaction between SP-A and LPS. In this regard, Kuzmenko and co-workers [265] suggested that SP-A distorts or perturbs membrane structure, creating defects that allow small hydrophilic molecules to enter and translocate through the bilayer. Such defects seem to be related to the formation by SP-A of extensive lattice-like structures on the surface of LPS-containing layers [211,266]. Pulmonary collectins also decrease the viability of *Histoplasma capsulatum* by increasing the permeability of the fungal cell wall in a calcium-dependent manner [267]. Although the precise mechanism involved in membrane permeabilization of this fungi is unknown, McCormack and co-workers [267] have suggested that membrane disruption could be due to the formation of calcium bridges between the collectins and fungal phospholipids. Growth of microorganism is also affected by the agglutinating activity of lung collectins, i.e., SP-D agglutinates *Candida albicans* [195,238] decreasing the availability of substrate to agglutinated yeasts [195].

On the other hand, a surfactant lipid mixture composed of DPPC, palmitoyloleoylphosphatidylcholine and palmitic acid has been shown to interfere with the interaction of non typable *Haemophilus influenzae* with pneumocytes by binding to the bacteria and preventing bacterial adhesion and internalization in alveolar epithelial cells [268]. In addition, these lipids can be endocytosed by pneumocytes by binding to the scavenger receptor class B type I, blocking bacterial uptake [268]. In vivo studies show that administration of the hydrophobic fraction of native surfactant, containing surfactant lipids and proteins SP-B and SP-C, significantly diminishes bacterial load in bronchoalveolar lavage and lung tissue of mice infected with this pathogen [268]. Although this antimicrobial activity has been ascribed to surfactant lipids, it is plausible that surfactant proteins SP-C and SP-B may play a bactericidal role against other pathogens. In this regard, bovine lung surfactant extract, a mixture of DPPC, palmitoyloleoylphosphatidylglycerol (POPG) and SP-B/-C, and a synthetic model lung surfactant, but not the lipids alone, all show antimicrobial activity against *E. coli* and *Staphylococcus aureus* [269]. SP-B and SP-C have a strong affinity for iron-regulated surface determinant proteins A and C [269], cell-wall receptors of *S. aureus* involved in the process of heme-iron acquisition. Binding to these receptors would disturb the bacterial surface, enhancing bacterial killing [269]. SP-B and SP-C also bind to β-barrel assembly machinery protein A and lipopolysaccharide transport protein D, two β-barrell proteins at the outer membrane of *E. coli,* lethally disrupting the bacterial outer membrane [269]. Additionally, incubation of *E. coli* with SP-C incorporated into liposomes of DPPC and POPG, but not the lipids alone, decreases bacterial growth by altering the membrane [269]. Although the mechanism by which SP-C disturbs the outer membrane is not clear, SP-C binding to LPS could be a key feature to disrupt the outer membrane and induce the release of LPS, rendering the cells permeable. This would facilitate the access of SP-C to the plasma membrane, where this protein would exert an antimicrobial activity [269]. The antimicrobial action of SP-B, which has been shown to aggregate and kill clinical isolates of *K. pneumoniae*, *P aeruginosa*, *S. aureus* and group B streptococcus by increasing membrane permeability [270], is inhibited by surfactant lipids, mainly POPG [270]. This suggests that endogenous SP-B may not play by itself a significant role in alveolar host defense. However, proSP-B, the SP-B precursor may be proteolitically cleaved into smaller peptide fragments that could retain its antimicrobial activity in the presence of surfactant lipids. In this regard, SP-B^N^, the N-terminal propeptide of SP-B (residues 31-191), which encodes a saposin-like domain, promotes the uptake of bacteria by macrophages and exerts a bactericidal effect at acidic pH, consistent with a lysosomal, antimicrobial function [271].

Binding of surfactant lipids and proteins to viruses results in enhanced phagocytosis and viral neutralization at the initial stages of infection [243,272]. Regarding lung collectins, the viral neutralizing activity of SP-D is enhanced compared to SP-A, playing an important role in the innate immune response to different viruses such as vaccinia or influenza A virus. The antiviral activity of lung collectins is related to their ability to bind to different proteins on the viral surface [243]. In general, interactions of lung collectins with viral proteins are calcium-dependent, involve the CRD and are facilitated by the multimerization of the full-length protein. The collagen domain of SP-A may also play a role in the antiviral action of the protein, since a recombinant variant of human SP-A composed of the neck and carbohydrate recognition domains can promote the replication of influenza A virus [273]. While SP-A prevents the recognition of influenza A virus by host cell receptors through binding of the sialylated asparagine 187 residue of SP-A to hemagglutinin [274,275], binding of SP-D to mannose-rich oligosaccharides close to the sialic acid-binding sites of hemagglutinin reduces viral uptake into epithelial cells [276,277], and sterically blocks the access of neuraminidase to surface-bound substrates [278,279] thereby preventing viral dissemination. This inhibitory effect has been related to the bulky collagen domain in the extended structure of dodecameric SP-D [279]. SP-A and SP-D also bind to the human immunodeficiency virus envelope glycoprotein 120 [280,281,282] and to the fusion and attachment glycoproteins of respiratory syncytial virus, F and G, respectively [283,284,285,286], neutralizing viral infectivity and inhibiting viral replication [281]. SP-A can mediate phagocytosis of herpes simplex virus type 1 by alveolar macrophages via its N-linked oligosaccharides [287]. To which viral surface protein(s) SP-A binds is not known. However, since SP-A binding to herpes simplex virus is inhibited by heparin, and heparin is known to bind to viral glycoproteins B and C, these proteins are likely candidates [287]. Other viral surface proteins recognized by lung collectins are the spike glycoprotein of the severe acute respiratory syndrome (SARS) coronavirus [288], the outer capsid glycoprotein 7 of non-enveloped rotavirus [289], and the A27 protein of vaccinia virus [290]. The potential interaction of surfactant proteins with proteins at the surface of the SARS-CoV-2 virus responsible of the emergent CoVid19 pandemic is currently under intensive scrutiny. Interactions of lung collectins with viral proteins can also result in deleterious effects during viral infections. In this regard, binding of SP-D to the glycoprotein of Ebola virus enhances infection in mammalian cells, facilitating the attachment of SP-D-bound virus to host cell co-receptors and probably affecting the inflammatory response [291]. On the other hand, the finding that constitutive expression of misfolded SP-C increases susceptibility to viral-induced cell death [292] suggests an antiviral activity for SP-C. This hypothesis is supported by the increased susceptibility of SP-C deficient mice to respiratory virus infection [235], which is reduced upon transgenic restoration of SP-C [197]. Surfactant lipids also play antiviral roles. Different molecular species of PG and PI have been shown to bind to respiratory syncytial virus and influenza A virus, inhibiting their interaction with the epithelial cell surface [231,293,294,295,296]. Neither the structural basis for lipid antagonism nor the inhibition mechanisms are known. It has been proposed that POPG and PI could act as decoy ligands for Toll-like receptors, given the structural similarities between the lipid A moiety of LPS and the anionic surfactant lipids [183]. Likewise, the structural similarities between anionic surfactant lipids and sialic acid containing lipid receptors for the hemagglutinin of H1N1 influenza A virus suggest that these lipids could also act as decoy receptors for viral attachment proteins [183].

The antimicrobial function of surfactant components may be modulated by interaction with other antimicrobial components of the alveolar fluid. Cooperative interactions of SP-A and SP-D with antimicrobial peptides have been suggested. For example, binding of SP-A to SP-B^N^ results in synergistic activity against SP-A– and SP-B^N^–resistant capsulated *K. pneumoniae* both in vitro and in vivo [253]. On the other hand, binding of SP-D to human neutrophil defensins results in antagonistic or cooperative effects on the hemagglutinin inhibitory and neutralizing activity of SP-D [297]. These effects depend on the viral strain and the multimeric forms of both proteins, with higher weight multimers precipitating SP-D in bronchoalveolar lavage fluid and thereby, reducing its hemagglutinin inhibitory activity [297]. On the contrary, combinations of SP-D with human β-defensins 5 and 6 show additive activity against influenza A virus [298]. SP-A, SP-D and the scavenger receptor-rich gp340 act cooperatively against influenza A virus, inducing viral aggregation and inhibiting hemagglutination [299]. Although both collectins bind to gp340 [249,250], this cooperative effect does not appear to require a direct interaction with gp340, at least for SP-D [299]. Whether this is also true for SP-A, needs to be clarified. Additionally, lung collectins can modulate the activity and toxicity of some antimicrobial peptides. By binding to β-defensin 3, SP-A negatively regulates the cytotoxicity of this peptide on epithelial cells without affecting its antimicrobial activity, protecting the lung epithelium from injury caused by an excess amount of β-defensin 3 liberated during inflammation [300].

## 6. Alveolar Epithelial Homeostasis

Surfactant proteins also contribute to the homeostasis of the alveolar epithelium by modulating apoptosis, promoting clearance of apoptotic cells, and enhancing tissue repair [15].

### 6.1. Modulation of Apoptosis

Alveolar epithelial apoptosis is an important contributor to the pathophysiology of lung diseases since severe alveolar epithelial apoptosis increases alveolar capillary permeability, and causes leakage of serum into alveoli and loss of compartmentalization with unrestricted mechanical transduction and signaling from the airways [301]. Excessive apoptosis may therefore affect the outcome of lung diseases, including adult respiratory distress syndrome [302], chronic obstructive pulmonary disease [303], and pulmonary fibrosis [304]. Therefore, a well-balanced interplay of cell apoptosis and proliferation is required to maintain the integrity and function of the lung.

Lung collectins have been shown to play a key role regulating apoptosis of alveolar epithelial cells apoptosis. In this regard, SP-D has been shown to decrease apoptosis both in vitro and in vivo. A recent study shows that SP-D-deficient mice have higher level of apoptotic cells in their alveolar spaces than wild type mice and intratracheal administration of recombinant SP-D reduces levels of apoptotic cells [263]. This reduction in apoptosis is due to a delayed activation of caspase 8 and 3 and externalization of phosphatidylserine [305], the aminophospholipid that in normal conditions is located in the inner leaflet of the plasma membrane, but flips to the outer leaflet in dying cells. Janssen et al. [306] have proposed that SP-A and SP-D suppress the phagocytic function of alveolar macrophages through tonic interaction with the transmembrane receptor signal inhibitory regulatory protein alfa (SIRPα) in the resting, noninflamed lung. Binding of SP-A or SP-D to SIRPα may facilitate the interaction of this receptor with cluster of differentiation CD47, and the subsequent negative control of phagocytosis by innate immune cells [307]. In type II cells, binding of SP-A to its receptor initiates a signaling pathway that regulates apoptosis through tyrosine phosphorylation and activation of phosphatidylinositol 3-kinase [308]. On the other hand, it has been recently demonstrated that the interaction of interleukin 8, a chemokine produced by macrophages and epithelial cells, with SP-A exacerbates cell damage in acute lung injury through inhibition of SP-A, promoting cell viability and suppressing apoptosis [309]. Interleukin 8 also binds to SP-B [309], inhibiting the protective effect of this protein on calcium ion transport. This would result in an increase of intracellular calcium ions that may induce passive depolymerization of cytoskeleton and destroy the endothelial barrier [310].

Regarding surfactant lipids, little is known about their role in apoptosis. Supplementation of Poractant alfa (Curosurf), an exogenous porcine-derived surfactant composed of surfactant lipids, SP-B, and SP-C, with POPG or dioleoylphosphatidylglycerol reduces alveolar epithelial cell apoptosis [311,312]. How these phospholipids regulate apoptosis is unknown, but they are supposed to act directly on alveolar epithelial cells [311]. Surfactant proteins SP-B and SP-C are not involved in principle in regulation of apoptosis. However, it has been shown that mutations in the SP-C gene produce aberrant forms of SP-C that can generate accumulation of aggregated forms of proSP-C, causing misfolded protein stress responses, inhibiting proteasome function, and activating caspase-3-mediated apoptosis [313].

### 6.2. Efferocytosis

Non-inflammatory removal of dying cells is important for maintenance of lung tissue homeostasis since apoptotic cells can undergo necrosis, releasing potentially damaging pro-inflammatory molecules and antigens that cause autoimmunity [314] and the eventual destruction of alveolar walls [315]. Lung collectins enhance the ingestion of apoptotic cells by alveolar macrophages [316,317], being SP-D a more potent promoter of apoptotic cell clearance than SP-A [316]. Removal of apoptotic cells by SP-A and SP-D occurs through simultaneous interaction with apoptotic cells and surface receptors on alveolar macrophages. The interaction of SP-A and SP-D with dying cells involves recognition of intracellular molecules that become exposed on the outside of the cell upon apoptosis. In this regard, both collectins have been shown to bind to DNA [318,319] and myeloperoxidase [320], a specific intracellular defence molecule of neutrophils that has been proposed to act as a bridging molecule between phosphatidylserine epitopes on apoptotic cells and SP-A, facilitating the internalization of dying cells by macrophages. On the other hand, the macrophage receptors calreticulin/CD91 and myosin 18A are involved in collectin-mediated clearance of dead cells [15,321]. Interestingly, the interactions of SP-A and SP-D with these receptors, as well as the binding of SP-D to DNA, occur through their collagenous domains [15,319,322,323]. Therefore, the potency of SP-D as promoter of apoptotic cell clearance may be linked to its large and extended collagen steams, which would facilitate the interaction between the macrophage receptors and apoptotic cells. This would allow the binding of calreticulin to dying cells and their subsequent targeting for removal by phagocytosis [324].

In vivo administration of Poractant alfa increases the clearance of apoptotic neutrophils [325]. Whether this effect is due to surfactant lipids or to surfactant proteins SP–B and/or –C is not clear. Although the mechanisms involved in this effect remain elusive, Willems and co-workers [325] have proposed that surfactant lipids could compete with SIRPα for binding SP-A and SP-D, thereby preventing their suppressive effects on phagocyte function.

### 6.3. Tissue Repair

Pathogens, damaged cells or toxic compounds may trigger an exacerbated or chronic inflammatory response that damages lung tissue, playing a critical role in the pathology and progression of different lung diseases such as emphysema [326] and lung fibrosis [327]. Resolution of inflammation and the subsequent regeneration of lung tissue requires replacement of injured cells by cells of the same type and the subsequent fibroplasia or fibrotic scarring in which connective tissue replaces normal parenchymal tissue, associated with an alternative, anti-inflammatory M2 polarization of macrophages [328,329,330]. Willems and co-workers demonstrated that pulmonary surfactant plays a role in the regeneration and remodeling of lung parenchyma [325]. SP-A has been shown to participate in the processes that control lung epithelial cell proliferation and apoptosis by binding to transforming growth factor β (TGF-β) [325,331], a potent inhibitor of epithelial cell proliferation. Formation of the SP-A/TGF-β complex protects TGF-β from inactivation by the latency-associated peptide [331] and facilitates TGF-β binding to its receptors, inducing signaling cascades involved in cell cycle progression and proliferation [127]. SP-A and SP-D also contribute to tissue repair by activating the polarization of alveolar macrophages to a M2 phenotype [323,332]. This effect has been related to the binding of the collagen domain of SP-A to myosin 18 [323]. This receptor, also known as SP-R210, forms a pro-inflammatory complex with CD14 and class A scavenger receptor (SR-A) that promotes internalization of LPS and subsequent signaling [333]. Therefore, SP-A may inhibit the SP-R210/CD14/SR-A complex, contributing to regulation and resolution of the inflammatory response [333]. In addition, through binding to interferon gamma (IFN-γ), SP-A suppresses IFN-γ interaction with its receptor, inhibiting the polarization of alveolar macrophages to a pro-inflammatory M1 phenotype [334] that has been shown to hinder fibroplasias [328].

On the other hand, SP-C [78] also plays a key role in maintaining alveolar integrity and repair since SP-C-null patients and animal models of SP-C deficiency develop fibrosis [105,335]. Moreover, mutations or decreased expression of the gene encoding SP-C, causes alveolar type II cell injury and aberrant repair of lung tissue to develop pulmonary fibrosis [335,336,337]. In this regard, Glasser and co-workers [234] showed that SP-C-deficient mice express markers of macrophage alternative activation associated with advancing pulmonary fibrosis. This suggests that SP-C negatively regulates the alternative activation of macrophages, preventing the development of fibrosis. Although the role of SP-C deficiency in fibrosis development has been documented by numerous lines of evidence, little is known about the interactions of SP-C with macrophage receptors involved in regulation of macrophage polarization.

## 7. Conclusions: Surfactant as a Hub in Alveolar Homeostasis

The extensive network of lipid–protein and protein–protein interactions described by this review to occur between lung surfactant molecules and host and pathogen cells at the alveolar spaces put surfactant structures at the center of lung homeostasis. Through these interactions, the surfactant network likely integrates signals from epithelial cells and the cells responsible for the innate and induced immune response, leading to a coordinated modulation of the respiratory scenario. This includes a proper control over an environment that needs to be at the same time protective against mechanical and biophysical demands and against infection, but relatively tolerant with the occasional entrance of exogenous particles and microorganisms inhaled within the thousands of liters of air breathed every day. On the other hand, lung surfactant-associated interactions and processes have to coordinate appropriate responses to challenges triggered by different physiopathological contexts, whose resolution defines the edge between health and illness. A full understanding of the different levels of complexity of the surfactant network will open unprecedented diagnostic and therapeutic opportunities.

## Figures and Tables

**Table 1:** Summary of protein/protein and protein/lipid interactions involved in alveolar homeostasis through regulation of surfactant film formation and stability, as well as surfactant levels in alveolar spaces.

Surfactant Protein	Interacting Protein or Lipid	Functional Role	References
**SP-A**	SP-B	Interfacial adsorption Secretion regulation * Cohesivity of multilayer reservoir *	[43,80,81,86] [30] [80]
	SP-B/PG	Interfacial adsorption Tubular myelin formation	[89] [24]
	DPPC	Lipid re-uptake to AE2C	[90]
	Cholesterol	Protection against inhibition	[83]
	PLA2	Lipid degradation inhibition	[91]
**SP-B**	SP-B	Interfacial adsorption (lipid transfer) Permeability Cohesivity (membrane stacking) LB assembly (membrane stacking)	[22,45,46,53] [59,66] [46,52] [52]
	SP-C	Interfacial adsorption Modulation of permeability and lipid transfer Pro SP-C processing *	[22,23] [59,66,68] [68]
	PG	Interfacial adsorption and film stability	[45,58,59,92]
	Cholesterol *	Unknown	[45]
**SP-C**	PG	Interfacial adsorption	[58]
	Cholesterol	Cholesterol removal from interfacial film (refining) Cholesterol removal from alveolar spaces * Crosstalk AE2C-AM	[47,69,77] [69] [78]
**SP-D**	PI	Regulation of re-uptake to AE2C	[27,93,94]
	GPR116 *	Regulation of secretion and re-uptake *	[95]

* Suggested potential interactions or roles.

## Abbreviations

ABCA1	ATP binding cassette subfamily A member 1
ABCA3	ATP binding cassette subfamily A member 3
ABCG3	ATP binding cassette subfamily G member 3
AE1C	alveolar epithelial type I cells
AE2C	alveolar epithelial type II cells
AM	alveolar macrophages
ARDS	acute respiratory distress syndrome
C1q	complement component 1q
CCT	cytidine triphosphate-phosphocoline cytidyltransferase
CD14	cluster of differentiation 14
CD91	cluster of differentiation 91
CRD	carbohydrate recognition domain
DPPC	dipalmitoylphosphatidylcholine
EMC3	endoplasmic reticulum membrane protein complex subunit 3
ER	endoplasmic reticulum
GM-CSF	granulocyte-macrophage colony stimulating factor
gp340	glycoprotein 340
GPR116	G-protein coupled receptor 116
IFN-γ	interferon gamma
LA	large aggregates
LB	lamellar bodies
LBP	lipopolysaccharide binding protein
LPCAT1	acyl CoA:lysophosphatidylcholine acyltransferase
LPS	lipopolysaccharide
MD2	myeloid differentiation factor 2
NETs	neutrophil extracellular traps
P63	tumor protein 63
PAP	pulmonary alveolar proteinosis
PC	phosphatidylcholine
PG	phosphatidylglycerol
PI	phosphatidylinositol
PLA2	phospholipase A2
POPG	palmitoyloleoylphosphatidylglycerol
RCT	reverse cholesterol transport
RDS	respiratory disease syndrome
SA	small aggregates
SARS	severe acute respiratory syndrome
SIRPα	signal inhibitory regulatory protein alfa
SNARE	*N*-ethylmaleimide-sensitive factor attachment receptor
SP-A	surfactant protein A
SP-B	surfactant protein B
SP-B^N^	*N*-terminal propeptide of SP-B
SP-C	surfactant protein C
SP-D	surfactant protein D
SP-R210	surfactant protein receptor 210
SR-A	class A scavenger receptor
STAR10	steroidogenic acute regulatory-related lipid transfer protein 10
TGF-β	transforming growth factor β
TLR4	Toll-like receptor 4
